# Impact of a kidney-adjusted ERAS^®^ protocol on postoperative outcomes in patients undergoing partial nephrectomy

**DOI:** 10.1007/s00423-024-03513-7

**Published:** 2024-10-23

**Authors:** Margarete Teresa Walach, Mona Körner, Christel Weiß, Tom Terboven, Julia Mühlbauer, Frederik Wessels, Thomas Stefan Worst, Karl-Friedrich Kowalewski, Maximilian Christian Kriegmair

**Affiliations:** 1 Department of Urology and Urosurgery, Medical Faculty Mannheim, University Medical Centre Mannheim, University of Heidelberg, Theodor-Kutzer-Ufer 1-3, 68167 Mannheim, Germany; 2 Department of Medical Statistics and Biomathematics, Medical Faculty Mannheim, University of Heidelberg, Mannheim, Germany; 3 Department of Anesthesiology, St. Josefskrankenhaus Heidelberg, Heidelberg, Germany; 4 German Cancer Research Center (DKFZ) Heidelberg, Division of Intelligent Systems and Robotics in Urology (ISRU), Heidelberg, Germany; 5 DKFZ Hector Cancer Institute at the University Medical Center Mannheim, Mannheim, Germany; 6 Urologische Klinik München Planegg, Planegg, Germany

**Keywords:** Renal cancer, Nephron sparing surgery, Partial nephrectomy, Enhanced recovery after surgery

## Abstract

**Purpose:**

Evaluation of a kidney-adjusted enhanced recovery after surgery (ERAS^®^) protocol (kERAS) in patients undergoing nephron-sparing surgery (PN).

**Methods:**

The kERAS protocol is a multidimensional protocol focusing on optimized perioperative fluid and nutrition management as well as strict intraoperative and postoperative blood pressure limits. It was applied in a prospective cohort (*n* = 147) of patients undergoing open or robotic PN. Patients were analyzed for the development of acute postoperative renal failure (AKI), achievement of TRIFECTA criteria, upstaging or new onset of chronic kidney disease (CKD) and length of hospital stay (LOS) and compared to a retrospective cohort (*n* = 162) without application of the protocol.

**Results:**

Cox regression analyses could not confirm a protective effect of kERAS on the development of AKI post-surgery. A positive effect was observed on TRIFECTA achievement (OR 2.2, 95% CI 1.0-4.5, *p* = 0.0374). Patients treated with the kERAS protocol showed less long-term CKD upstaging compared to those treated with the standard protocol (*p* = 0.0033). There was no significant effect on LOS and new onset of CKD.

**Conclusion:**

The implementation of a kERAS protocol can have a positive influence on long-term renal function in patients undergoing PN. It can be used safely without promoting AKI. Furthermore, it can be realized with a manageable amount of additional effort.

**Supplementary Information:**

The online version contains supplementary material available at 10.1007/s00423-024-03513-7.

## Introduction

The implementation of an enhanced recovery after surgery (ERAS^®^) protocol is an important approach to multimodal, multidisciplinary and modern care of surgical patients and widespread among surgical specialties [1]. ERAS^®^ protocols are designed to achieve early recovery after surgical procedures by maintaining pre-surgical organ function and reducing the stress response to improve outcomes [2]. Key elements are standardized analgesic and anesthetic regimes, optimization of nutrition and fluid management, avoidance of perioperative fasting, carbohydrate loading preoperatively and early mobilization [3–5]. These factors modify the physiological response to the stress of major surgery and lead to an earlier return of bowel function, a faster resumption of daily activities and a reduction of complications and of hospital stay and readmission [6]. Consequently, health costs can be lowered [7, 8]. Although much of the data arise from colorectal surgeries, multiple elements of the ERAS^®^ perioperative care pathways are applicable to major urological surgery and the number of reports and ongoing trials on the use of ERAS^®^ in urological surgery is rising [9–14]. The use of ERAS^®^ protocols for radical cystectomy (RC) is a routine practice in most high volume medical centers and the ERAS^®^ society has even published guidelines for patients undergoing RC [15]. However, data on the use of ERAS^®^ programs in kidney surgery is rare, even though there are overall complication rates up to 25% in patients undergoing partial nephrectomy (PN) and the risk of postoperative acute kidney injury (AKI) and chronic kidney disease (CKD) [16–21].

Presumed renoprotective agents such as mannitol, allopurinol or sildenafil have been studied to limit postoperative loss of renal function, but the evidence for their use is ambiguous and no clinical benefit has been demonstrated, so their use has not become established in clinical routine [22–24]. Therefore, assessment and implementation of a kidney-adjusted ERAS^®^ protocol (kERAS) seems judicious. Thus, this study aimed to evaluate the effect of a newly developed kERAS protocol, including improved fluid management, carbohydrate loading and strict kidney-adapted blood pressure control on postoperative acute and long-term renal function outcomes, TRIFECTA achievement and length of hospital stay (LOS).

## Materials and methods

### Study population and data collection

Patients with renal masses undergoing open or robot-assisted nephron sparing surgery at our high volume university medical center between 01/2013 and 08/2020 were included. Of a total of 690 patients, data of 309 patients was available for analysis. The patient cohort was divided into two groups: a retrospective and a prospective cohort. Data collection was performed using medical records, laboratory investigations and radiological reports. Long-term renal function parameters were determined by contacting the treating physicians (urologists and general practitioners). Patients with single kidneys, with renal shrinkage or with previous PN were excluded. Flow charts of the exclusion criteria are illustrated in Supplementary Figs. 1 and 2.

### Perioperative kidney-adjusted ERAS^®^ protocol

The prospective patient cohort received a kidney-adapted perioperative treatment in accordance with established renoprotective treatment strategies [25]. Optimized perioperative fluid management consisted of the application of intravenous balanced fluid overnight (1 ml/kg/h) before surgery until transportation in the operating room and a balanced fluid management intraoperatively (crystalloid fluid 2 mg/kg/h). Maintaining a delicate balance between avoiding hypovolemia and preventing fluid overload, using balanced crystalloids, goal-directed fluid therapy, and vasopressor support as needed, while closely monitoring hemodynamics, helps ensure stable renal perfusion, minimize the risk of AKI, and promote optimal outcomes for the patient.

Carbohydrate loading was another component of the kERAS protocol including the administration of 200 ml of an isotonic, 21% carbohydrate containing clear fluid the evening before surgery, another 200 ml one hour before transfer in the operating room, one carbohydrate drink after arrival in the postanaesthesia recovery room and three carbohydrate drinks on the first postoperative day. Carbohydrate loading before PN enhances recovery by improving glycemic control, preserving muscle mass, reducing catabolism, and promoting faster return of gastrointestinal function [3].

The third main aspect of the kERAS protocol was a strict intra- and postoperative blood pressure management with intraoperative mean arterial pressure (MAP) goals of ≥ 70 mmHg (or ≥ 80 mmHg if arterial hypertension was known). Intraoperative hypotension lacks a clear definition, leading to varying thresholds and its interpretation [26, 27]. In our study, we set a target MAP of ≥ 70 mmHg, based on expert recommendations from anesthesiologists and nephrologists, and supported by existing literature on renal impairment. Avoiding intraoperative MAP drops below 70 mmHg is crucial to prevent renal hypoperfusion and potential kidney damage, as low MAP is linked to acute kidney injury, myocardial injury, and even death [28–31].

Further aspects are summarized in Supplementary Fig. 3.

### Outcome measures

The primary outcome measure of this study was the development of AKI within seven days after surgery. Secondary outcome measures were TRIFECTA achievement, CKD upstaging or new onset of CKD at 9–15 months post-surgery and LOS. AKI and CKD were evaluated according to the international guidelines for kidney disease (KDIGO criteria) [32]. TRIFECTA achievement was defined according to Hung et al. (negative surgical margin, minimal renal function decrease (eGFR decrease postoperative ≤ 10%), no complications) [33]. Achievement of the TRIFECTA was considered to have been reached if all criteria were met.

### Statistical analysis

Descriptive characteristics of the cohorts were analyzed as follows: Frequencies and proportions were assessed for categorical variables, while means, standard deviations, medians and interquartile ranges (IQR) were calculated for continuous variables. Comparisons between groups were performed using X^2^ or Fisher’s exact test for categorical data, Cochran-Armitage Trend Test for ordinal data, Mann-Whitney U test to compare medians of non-normally distributed continuous data and Two-Sample T-test to compare means. All tests for comparing two groups were two-sided. Testing for presence of a trend was performed whenever applicable. Uni- and multivariable logistic regression analyses (backward selection) were performed to evaluate the association of the kERAS protocol, patient, tumor and surgical characteristics and the primary and secondary outcomes. Statistical significance level was set to α = 0.05. All statistical calculations were done using the software SAS^®^ (SAS Institute Inc., Cary, North Carolina, USA), version 9.4.

## Results

The retrospective cohort (*n* = 162) consisted of patients who received standard perioperative treatment (until 2018) and the prospective cohort (*n* = 147) consisted of patients who were treated with our kERAS protocol (since 2019). A significant difference between the patient characteristics of both study cohorts could be determined regarding age (retrospective cohort 61.1 ± 12.8, prospective cohort 66.2 ± 11.2, *p* = 0.0002) and CKD stages in the cohorts (trend tests showed significantly more patients with higher stages of CKD in the prospective cohort, *p* = 0.0333). The baseline patient and tumor characteristics of the two study groups are illustrated in Table 1.

**Table 1:** Patient and tumor characteristics of the retrospective and prospective study cohort

	All patients	Retrospective cohort	Prospective cohort	*p*-value
Number of patients (n)	309	162	147	
Age in years, mean ± SD	63.5 ± 12.3	61.1 ± 12.8	66.2 ± 11.2	**0.0002**
Gender, n (%)				0.5310
Male Female	209 (67.6) 100 (32.4)	107 (66.1) 55 (33.9)	102 (69.4) 45 (30.6)	
BMI (kg/m2), median (IQR)	27.1 (24.3–30.7)	26.9 (24.3–30.7)*	27.1 (24.3–31.1)	0.2817
ASA				0.4287
≥ 3 < 3	88 (28.5) 221 (71.5)	43 (26.5) 119 (73.5)	45 (30.6) 102 (69.4)	
RENAL score, n (%)				0.2795
Low complexity Moderate complexity High complexity	85 (27.9) 176 (57.7) 44 (14.4)	42 (26.1)* 91 (56.5)* 28 (17.4)*	43 (29.9)^+^ 85 (59.0)^+^ 16 (11.1)^+^	
Tumor size (cm), median (IQR)	3.1 (2.4–4.5)	3.1 (2.5–4.5)	3.1 (2.3–4.4)*	0.7153
Tumor side, n (%)				0.1569
Left Right	153 (49.5) 156 (50.5)	74 (45.7) 88 (54.3)	79 (53.7) 68 (46.3)	
Pathologic T stage, n (%)				0.2064
pT1a pT1b pT2a pT2b ≥pT3a	150 (61.2) 67 (27.3) 9 (3.7) 0 (0.0) 19 (7.8)	83 (63.4)* 35 (26.7)* 6 (4.6)* 0 (0.0)* 7 (5.3)*	67 (58.8) 32 (28.1) 3 (2.6) 0 (0.0) 12 (10.5)	
Malignant histology, n (%)	247 (79.9)	132 (81.5)	115 (78.2)	0.4762
Histological of malignant tumors, n (%)				0.0501
Clear cell Papillary type Chromophobe Others	157 (63.6) 58 (23.5) 22 (8.9) 10 (4.0)	91 (68.9) 24 (18.2) 14 (10.6) 3 (2.3)	66 (57.4) 34 (29.6) 8 (7.0) 7 (6.1)	
Pathological WHO/ISUP grading, n (%)				0.0586
G1 G2 G3 G4	114 (49.8) 112 (48.9) 3 (1.3) 0 (0.0)	68 (55.7) 53 (43.4) 1 (0.8) 0 (0.0)	46 (43.0)^•^ 59 (55.1)^•^ 2 (1.9)^•^ 0 (0.0)^•^	
Arterial hypertension, n (%)	202 (65.4)	100 (61.7)	102 (69.4)	0.1576
Diabetes mellitus, n (%)	50 (16.2)	21 (13.0)	29 (19.7)	0.1068
Chronic kidney disease stage, n (%)^#^				**0.0333**
Stage 2 Stage 3a Stage 3b Stage 4 Stage 5	142 (46.0) 43 (13.9) 25 (8.1) 6 (1.9) 1 (0.3)	73 (45.1) 23 (14.2) 6 (3.7) 1 (0.6) 1 (0.6)	69 (46.9) 20 (13.6) 19 (12.9) 5 (3.4) 0 (0.0)	

*data of 1 patient missing, ^+^data of 3 patients missing, ^∞^data of 6 patients missing, ^•^data of 4 patients missing, ^#^Stage 1 not included as corresponding to normal kidney function; SD = standard deviation, BMI = body mass index, ASA = American Society of Anesthesiologists, IQR = interquartile range, n.c. = not calculable, WHO = World Health Organization, ISUP = International Society of Urologic Pathology

Surgical approach showed more patients undergoing open surgery in the retrospective cohort while more patients in the prospective cohort underwent RAPN (*p* = 0.0028). In the retrospective cohort more patients underwent PN applying renal ischemia (143 (88.3%) vs. 109 (74.1%), *p* = 0.0014) and ischemia duration was longer in this cohort (18 (14–22) vs. 15 (13–19), *p* = 0.0036). The mean MAP was lower in the retrospective cohort (77.9 ± 6.2 mmHg vs. 80.1 ± 7.0, *p* = 0.0020) and more patients showed a MAP < 70 mmHg at least once during surgery in this cohort (108 (66.7%) vs. 78 (53.1%), *p* = 0.0147). Furthermore, the study groups differed in baseline eGFR (retrospective cohort 81 (65–95) ml/min/1.73m^2^ vs. prospective cohort 71 (53–88) ml/min/1.73m^2^, *p* < 0.0001), which was determined on average 7 days before surgery. The eGFR dropped significantly in the retrospective cohort compared to the prospective cohort and compared to the to the baseline eGFR levels on different time points in the postoperative course. No difference regarding the development of a postoperative AKI, and new onset CKD at 9–15 months postoperative could be observed. Nonetheless, in the retrospective cohort, CKD upstaging was significantly more frequent (*p* = 0.0033). All results are shown in Table 2.

**Table 2:** Surgical characteristics, postoperative outcomes and follow-up

	All patients	Retrospective cohort	Prospective cohort	*p*-value
Surgical approach, n (%)				**0.0028**
OPN RAPN	232 (75.1) 77 (24.9)	133 (82.1) 29 (17.9)	99 (67.3) 48 (32.7)	
Surgery duration (min), median (IQR)	140 (116–168)	140.5 (117–167)	140 (115–170)	0.9304
Ischemia, n (%)	252 (81.6)	143 (88.3)	109 (74.1)	**0.0014**
Ischemia duration (min), median (IQR)	16.5 (13–20)	18 (14–22)	15 (13–19)	**0.0036**
Intraoperative MAP (mmHg), mean ± SD	79.0 ± 6.7	77.9 ± 6.2	80.1 ± 7.0	**0.0020**
Episode of MAP < 70 mmHg, n (%)	186 (60.2)	108 (66.7)	78 (53.1)	**0.0147**
Use of epidural catheter, n (%)	192 (62.1)	110 (67.9)	82 (55.8)	**0.0283**
Blood loss (ml), median (IQR)	200 (150–400)	200 (100–350)*	250 (150–500)^∞^	0.0523
Positive surgical margin, n (%)	10 (4.1)	4 (3.1)^∞^	6 (5.3)^∞^	0.5208
Transfusion, n (%)	26 (8.4)	15 (9.3)	11 (7.5)	0.5743
Clavien-Dindo grade ≥ 3, n (%)	43 (13.9)	27 (16.7)	16 (10.9)	0.1425
Length of stay (days), median (IQR)	7 (6–9)	7 (6–8)	7 (6–9)	0.7149
TRIFECTA achievement, n (%)	44 (18.1)	18 (13.8)^∞^	26 (23.0)^∞^	0.0643
Baseline Hb-level (g/dl), median (IQR)	14.2 (13.3–15.3)	14.2 (13.3–15.2)	14.3 (13.3–15.4)	0.7234
Baseline creatinine (mg/dl), median (IQR)	0.93 (0.81–1.09)	0.92 (0.79–1.05)	0.95 (0.83–1.16)	0.0578
eGFR (ml/min/1.73m^2^), median (IQR)				
Baseline First day postoperative 3rd day postoperative 7th day postoperative 2–4 months postoperative 5–8 months postoperative 9–15 months postoperative 16–20 months postoperative >20 months postoperative	77 (60–92) 53 (43–69) 55 (39–72) 43 (32–61) 67 (52–85) 65 (48–86) 68.5 (54–85) 67.5 (53.5–84) 69 (52–83)	81 (65–95) 53 (43–66)^ϕ^ 57 (44–71)^•^ 51 (41–81)^#^ 71 (55-88.5)^∞∞^ 69.5 (54–89)^ϕϕ^ 67 (54.5–85)^••^ 67 (55–86)^##^ 72 (55.5–85)^∞∞∞^	71 (53–88) 53.5 (42-69.5)^¤^ 52.5 (38–72)^§^ 35 (29.5–52)** 64 (47–84)^∞∞^ 63 (46–83)^¤¤^ 71.5 (52–85)^§§^ 69.5 (53–83)*** 65 (48-78.5)^∞∞∞^	**< 0.0001** 0.9529 0.3298 **0.0071** **0.0363** 0.0827 0.9454 0.6447 **0.0122**
eGFR drop (ml/min/1.73m^2^), median (IQR)				
Preop-first day Preop-3rd day Preop-7th day Preop-2-4 months postoperative Preop-5-8 months postoperative Preop-9-15 months postoperative Preop-16-20 months postoperative	15 (5–27) 15 (6–26) 10 (3–22) 3 (-3 - ∞9) 4 (-3 - ∞10) 3 (-3 - ∞9) 3 (-5.5 - ∞10)	22 (9–33)^ϕ^ 22 (10–30)^•^ 12 (6–20)^#^ 5 (-1 - ∞14.5)^∞∞^ 7.5 (-1 - ∞15)^ϕϕ^ 5 (-1 - ∞17)^••^ 4.5 (-1 - ∞16)^##^	11 (1.5–21)^¤^ 11 (5–20)^§^ 9 (2–23)** 1 (-7 - ∞5)^∞∞^ 2 (-6 - ∞8)^¤¤^ 1 (-5 - ∞7)^§§^ 1 (-7 - ∞8)***	**< 0.0001** **0.0009** 0.5153 **0.0104** **0.0028** **0.0002** **0.0058**
Intraoperative urine output (ml), median (IQR)	500 (300–800)	450 (300–700)^¤^	500 (300–800)^¤^	0.3177
Intraoperative fluid balancing (ml), median (IQR)	3000 (2028–3500)	3000 (2510–4000)	2500 (2000–3027)	**< 0.0001**
Use of medication increasing blood pressure during surgery, n (%)	295 (95.5)	154 (95.1)	141 (95.9)	0.7177
Use of medication decreasing blood pressure during surgery, n (%)	172 (55.7)	111 (68.5)	61 (41.5)	**< 0.0001**
AKI, n (%)	150 (48.5)	78 (48.1)	72 (49.0)	0.8839
CKD upstaging at 9-15th months post-surgery, n (%)	19 (13.3)	14 (23)^ϕϕϕ^	5 (6.1)^¤¤¤^	**0.0033**
New onset CKD at 9-15th months post-surgery, n (%)	23 (53.5)	12 (52.2)^•••^	11 (55)^§§§^	0.8530

*data of 10 patients missing, ^∞^data of 5 patients missing, ^∞^data of 2 patients missing, ^ϕ^data of 59 patients missing, ^¤^data of 7 patients missing, ^•^data of 99 patients missing, ^§^data of 33 patients missing, ^#^data of 137 patients missing, **data of 119 patients missing, ^∞∞^data of 94 patients missing, ^∞∞^data of 80 patients missing, ^ϕϕ^data of 96 patients missing, ^¤¤^data of 66 patients missing, ^••^data of 78 patients missing, ^§§^data of 45 patients missing, ^##^data of 100 patients missing, ***data of 73 patients missing, ^∞∞∞^data of 46 patients missing, ^∞∞∞^data of 67 patients missing, ^ϕϕϕ^data of 43 patients missing, ^¤¤¤^data of 31 patients missing, ^•••^data of 35 patients missing, ^§§§^data of 14 patients missing; OPN = open partial nephrectomy, RAPN = robot-assisted partial nephrectomy, IQR = interquartile range, WIT = warm ischemia time, MAP = mean arterial pressure, SD = standard deviation, Hb = hemoglobin, eGFR = estimated glomerular filtration rate, AKI = acute kidney injury, CKD = chronic kidney disease

**Fig. 1:**
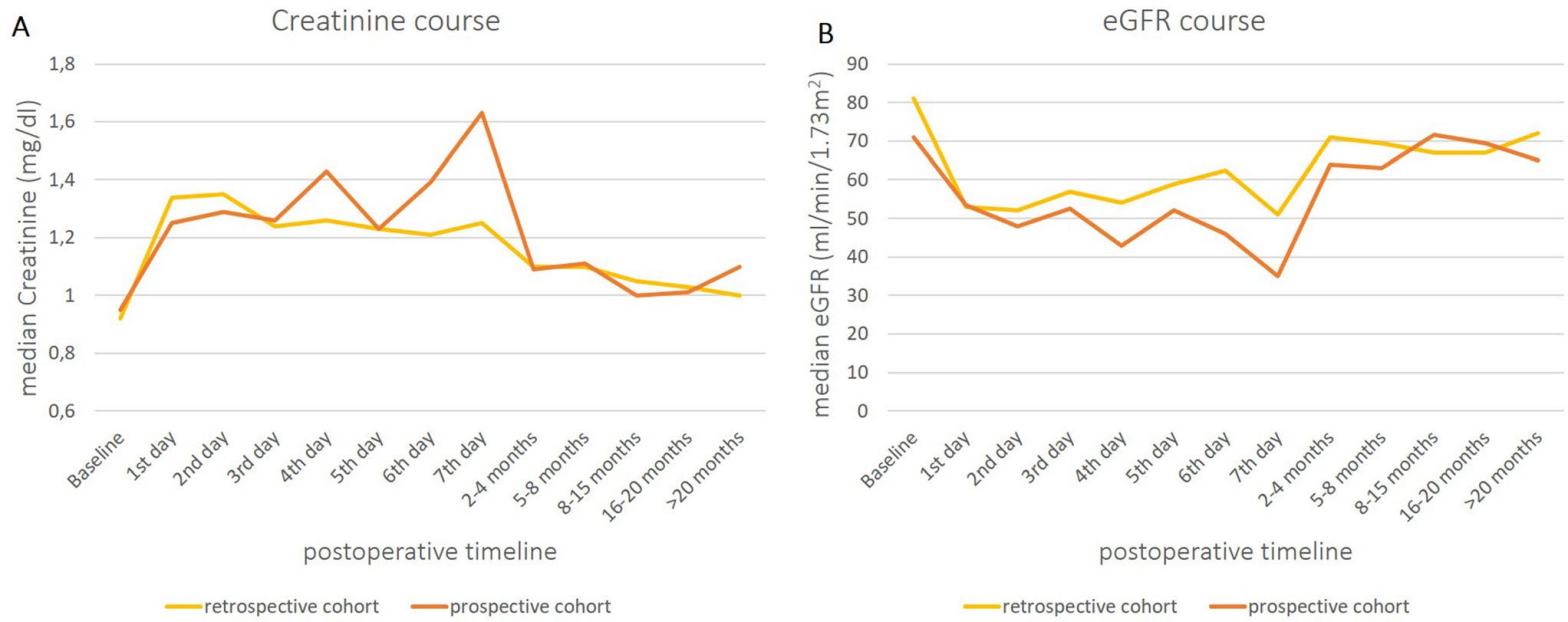
eGFR and Creatinine levels at different time points in the postoperative course

Creatinine levels and eGFR differed between both study groups at diverse time points in the postoperative course. Results are visualized in Fig. 1.

In the univariable logistic regression analyses male gender (*p* = 0.0011), surgery time (*p* = 0.0203), arterial hypertension (*p* < 0.0001), episodes of MAP < 70 mmHg during surgery (*p* = 0.0131) and RENAL score (*p* = 0.0079) showed to be significantly associated with the development of AKI. However, usage of the kERAS protocol did not show to have a protective impact on the development of postoperative AKI. In the multivariable logistic regression analysis male gender (*p* = 0.0045), arterial hypertension (*p* = 0.0002), episodes of MAP < 70 mmHg during surgery (*p* = 0.0464) and RENAL score (*p* = 0.0041) remained independent predictors for the development of AKI. Results of the uni- and multivariable logistic regression analyses regarding the primary endpoint AKI are shown in Table 3.

**Table 3:** Uni- and multivariable analyses for the development of AKI

Covariates	AKI					
	Univariable analysis			Multivariable analysis		
	OR	95% CI	*p*-value	OR	95% CI	*p*-value
Gender (male: female)	2.272	1.388–3.720	**0.0011**	2.147	1.267–3.639	**0.0045**
Age (per year)	1.005	0.987–1.024	0.5775			
WIT ≥ 25 min	1.266	0.618–2.591	0.5185			
Surgery time (per hour)	1.500	1.065–2.112	**0.0203**			0.2383
Arterial hypertension	2.719	1.665–4.441	**< 0.0001**	2.676	1.591–4.503	**0.0002**
Congestive heart disease	1.223	0.432–3.460	0.7041			
Preoperative CKD	1.336	0.818–2.183	0.2467			
Diabetes mellitus	1.072	0.585–1.964	0.8219			
Episode(s) of MAP < 70 mmHg	1.794	1.130–2.849	**0.0131**	1.650	1.008–2.702	**0.0464**
RENAL per grade						
Low Intermediate High	1.636	1.138–2.352	**0.0079**	1.773	1.199–2.620	**0.0041**
ASA score ≥ 3	1.592	0.968–2.620	0.0672			0.8840
Postoperative bleeding (transfusion and/or aneurysm)	1.591	0.817–3.097	0.1717			
Use of ERAS kidney protocol	1.034	0.661–1616	0.8839			

AKI = acute kidney injury, OR = odds ratio, CI = confidence interval, WIT = warm ischemia time, MAP = mean arterial pressure, ASA = American Society of Anesthesiologists

The results of the secondary endpoints are displayed in Table 4. In the multivariable logistic regression analysis surgery time (*p* = 0.0065), absence of arterial hypertension (0.0003), lower RENAL score (*p* = 0.0128) and the use of the kERAS protocol (*p* = 0.0374) showed to be independent predictors of TRIFECTA achievement. Regarding new onset CKD at 9–15 months post-surgery and/or CKD upstaging, only age (*p* = 0.0019) and surgery time (*p* = 0.284) showed to have independent prognostic significance. Multivariable analyses revealed that age (*p* = 0.0112), RENAL score (*p* = 0.0013), ASA score (*p* = 0.0008) and postoperative bleeding (*p* < 0.0001) have significant independent prognostic influence on LOS.

**Table 4:** Uni- and multivariable analyses for the secondary endpoints

Covariates	TRIFECTA achievement						New onset CKD at 9–15 months post-surgery or CKD upstaging						Length of hospital stay (more than 7 days)					
	Univariate analysis			Multivariate analysis			Univariate analysis			Multivariate analysis			Univariate analysis			Multivariate analysis		
	OR	95% CI	*p*-value	OR	95% CI	*p*-value	OR	95% CI	*p*-value	OR	95% CI	*p*-value	OR	95% CI	*p*-value	OR	95% CI	*p*-value
Gender (male: female)	0.702	0.349–1.410	0.3200				0.879	0.608–5.807	0.2734				0.929	0.572–1.510	0.7662			
Age (per year)	0.977	0.952–1.003	0.0845	--	--	0.7031	1.094	1.031–1.161	**0.0031**	0.904	0.848–0.963	**0.0019**	1.030	1.010–1.051	**0.0029**	1.029	1.007–1.052	**0.0112**
Surgery time (per hour)	0.444	0.252–0.780	**0.0048**	0.986	0.977–0.996	**0.0065**	2.554	0.999–6.530	0.0502	0.319	0.115–0.886	**0.0284**	1.512	1.071–2.134	**0.0188**	--	--	0.1041
Arterial hypertension	0.335	0.171–0.653	**0.0013**	0.253	0.120–0.530	**0.0003**	4.200	1.335–13.210	**0.0141**	--	--	0.2953	1.319	0.813–2.142	0.2625			
Preoperative CKD	0.538	0.273–1.059	0.0728	--	--	0.2289	nc	--	--				1.550	0.929–2.587	0.0935	--	--	0.9469
Episode(s) of MAP < 70 mmHg	0.412	0.212–0.801	**0.0089**	--	--	0.1107	0.558	0.156–1.998	0.3698				1.488	0.928–2.385	0.0991	--	--	0.1789
RENAL (per grade)																		
Low Intermediate High	0.534	0.313–0.912	**0.0216**	0.477	0.266–0.854	**0.0128**	0.950	0.420–2.148	0.9023				1.819	1.250–2.645	**0.0018**	1.974	1.306–2.985	**0.0013**
ASA score ≥ 3	0.641	0.289–1.418	0.2719				2.540	0.632–10.207	0.1889				2.852	1.716–4.741	**< 0.0001**	0.376	0.212–0.665	**0.0008**
Postoperative bleeding (transfusion and/or aneurysm)	nc	--	--				5.182	0.609–44.117	0.1322				6.916	3.165–15.110	**< 0.0001**	7.171	3.097–16.604	**< 0.0001**
Use of ERAS kidney protocol	1.860	0.958–3.609	0.0667	2.175	1.046–4.521	**0.0374**	0.752	0.256–2.212	0.6048				1.346	0.852–2.126	0.2022			

CKD = chronic kidney disease, OR = odds ratio, CI = confidence interval, MAP = mean arterial pressure, ASA = American Society of Anesthesiologists, nc = not calculable

## Discussion

ERAS^®^ protocols are well studied in numerous surgical specialties and are widely used, especially in major surgery. In contrast, evidence on potential benefits of using ERAS^®^ protocols for patients undergoing PN is sparse. In this study, we aimed to evaluate the value and impact of a kERAS protocol on the postoperative renal function, on TRIFECTA achievement and on LOS in patients undergoing PN.

Using our kERAS protocol, we did not observe a significant reduction in postoperative AKI compared to the standard protocol. Both cohorts had similar rates of sudden postoperative renal function decline after PN. This finding aligns with a meta-analysis of 19 studies involving 17,205 patients undergoing major surgery, which found no beneficial effect of ERA^®^S protocols on AKI incidence compared to standard care [34]. As more specialties have adopted ERAS^®^ protocols, concerns have been raised that these protocols, which include varying fluid management strategies, multimodal analgesia with non-steroidal anti-inflammatory drugs, and the use of vasoactive drugs, may actually promote the development of postoperative AKI [35–37]. However, it should be noted, that there is a narrow range for optimal intraoperative fluid therapy and suboptimal care in any of the relevant phases (preoperative, intraoperative, and postoperative) may consequently undermine best practice within the rest of the ERAS^®^ pathway [38]. Thus, differences in fluid management and perioperative blood pressure management may introduce bias and make comparisons between studies difficult. Optimized ERAS^®^ protocols with special consideration of renal function might yield different results. Although we could not recognize a significant protective effect of our kERAS protocol on AKI, our primary endpoint results confirm the protocol’s safety. Several other studies have also found no increase in the rate of postoperative AKI after implementation of an ERAS^®^ protocol [39–41]. Furthermore, although the proportion of pre-existing CKD was higher in our prospective group and the patients were older, both of which are known risk factors for the development of postoperative AKI, the rate was the same in both cohorts, suggesting some protective influence of the ERAS^®^ protocol on postoperative renal function [42, 43].

In our study, we observed less long-term upstaging of pre-existing CKD in the prospective cohort, possibly suggesting a beneficial effect of kERAS in patients with known impaired renal function. Postoperative renal insufficiency is known to be a significant predictor of cardiovascular-specific and overall survival, and patients are more likely to die of complications related to CKD than those related to the RCC itself [44, 45]. Thus, from a clinical perspective long-term renal function may be one of the most important determinants of postoperative morbidity and mortality and renoprotective strategies are therefore essential. Regarding new onset of CKD at 9–15 months post-surgery, no difference was observed between the two cohorts. However, as the groups differed significantly in age, with older patients in the prospective cohort, which is physiologically associated with poorer renal function and reflected in a worse baseline GFR in the prospective group. The protective influence of ERAS^®^ may therefore be underestimated in our cohort and might be more pronounced in groups with similar age and preoperative GFR levels.

Furthermore, we observed a positive impact of the kERAS protocol on TRIFECTA achievement in the prospective cohort. This secondary endpoint showed a significant result even though we used probably the strictest definition of TRIFECTA achievement: negative surgical margin, minimal decline in renal function (eGFR decrease postoperative ≤ 10%), no complications [33]. This is consistent with a study comparing standard management and an ERAS^®^ protocol in open radical cystectomy showing higher rates of TRIFECTA achievement in the ERAS^®^ cohort [46].

We assume that the positive influence of our kERAS protocol on TRIFECTA achievement may be attributed to a reduced perioperative stress response and better conditions to undergo surgery with regard to kidney function. We were unable to determine from our study which specific components of the kERAS protocol had the most significant impact on the individual elements of the TRIFECTA criteria. Further research would be needed to explore the effects of each component separately.

In addition to the kERAS protocol other influencing factors regarding TRIFECTA achievement were arterial hypertension, RENAL score and duration of surgery.

The use of the kERAS protocol did not show a protective effect on LOS after PN. In contrast to this result, Dunkman et al. were able to record a significant reduction in LOS in patients undergoing open radical cystectomy [47]. This discrepancy might be probably due to a reduction in postoperative ileus after radical cystectomy, which is known to be a major driver of LOS. As postoperative ileus is not a common complication of PN, this effect is not predominantly present in patients after PN and therefore not reflected in LOS. Known risk factors, such as age, RENAL score, ASA score and transfusion were significant negative influencing factors for LOS after PN.

Our study’s findings must be interpreted in light of its limitations. While kERAS cohort data were collected prospectively, retrospective cohort data relied on stored patient records, which may lack protection against confounding due to selection bias typically provided by randomization.

The variables selected as possible predictors were based on the available literature and investigator hypotheses, but other factors may have played a role. We did not determine readmission rates, which could offer interesting additional insights. Multiple missing values of long-term renal function could jeopardize the validity of our results, and a longer follow-up may yield more meaningful results. Additionally, our analysis included only patients with bilateral kidneys, potentially reducing accuracy in baseline creatinine adjustment compared to patients with single kidneys. We also did not assess if patients had a functional single kidney. Finally, ERAS^®^ protocols like ours consist of various components, making it difficult to identify which interventions are most impactful.

To our knowledge, this is the first study to evaluate the impact of a kidney-adjusted ERAS^®^ protocol in patients undergoing PN. Based on our results, we are convinced that the implementation of a kERAS protocol can have a positive impact on postoperative outcome and can be realized with a manageable amount of additional effort. Our results highlight the benefit of implementing such a protocol in patients, especially those with pre-existing impaired renal function, undergoing PN.

## Conclusion

In this study, the impact of a kidney-adjusted ERAS protocol was evaluated for the first time and it showed a positive effect on individual aspects of the postoperative outcome, such as TRIFECTA achievement and prevention from CKD upstaging, after renal-sparing surgery. Although certain components of the protocol may not have a measurable effect on their own, the use of the protocol has been shown to have a positive effect on long-term renal function, especially in patients with pre-existing CKD, and to be safe to use. On the other hand, the kERAS protocol did not significantly reduce the development of AKI compared to the standard protocol. This may be due to the severe acute surgical damage, resulting in AKI in some patients, which could not be adequately prevented by our optimized perioperative management. Future studies are needed to determine whether protocol modifications could further reduce rates of AKI and development of long-term CKD in patients undergoing PN. More specific patient groups, stratified by the severity of pre-existing CKD and characteristics such as single kidneys, in an exclusively prospective design or a matched-pair analysis could further elucidate the impact of the protocol. At the same time, the individual components of the protocol should be analyzed in order to work out which components have the greatest influence on the perioperative outcome.

## Electronic supplementary material

Below is the link to the electronic supplementary material.

Supplementary Fig. 1: Flow chart of exclusion criteria and cohort size of the retrospective cohort

Supplementary Fig. 2: Flow chart of exclusion criteria and cohort size of the prospective cohort

Supplementary Fig. 3: Components of the kidney-adjusted ERAS protocol

## Data Availability

No datasets were generated or analyzed during the current study.
