# Supplementary figures and images for: Impact of a kidney-adjusted ERAS® protocol on postoperative outcomes in patients undergoing partial nephrectomy
Source: Langenbecks Arch Surg. 2024 Oct 23;409(1):319. doi: 10.1007/s00423-024-03513-7 (PMC11499443; doi:10.1007/s00423-024-03513-7)

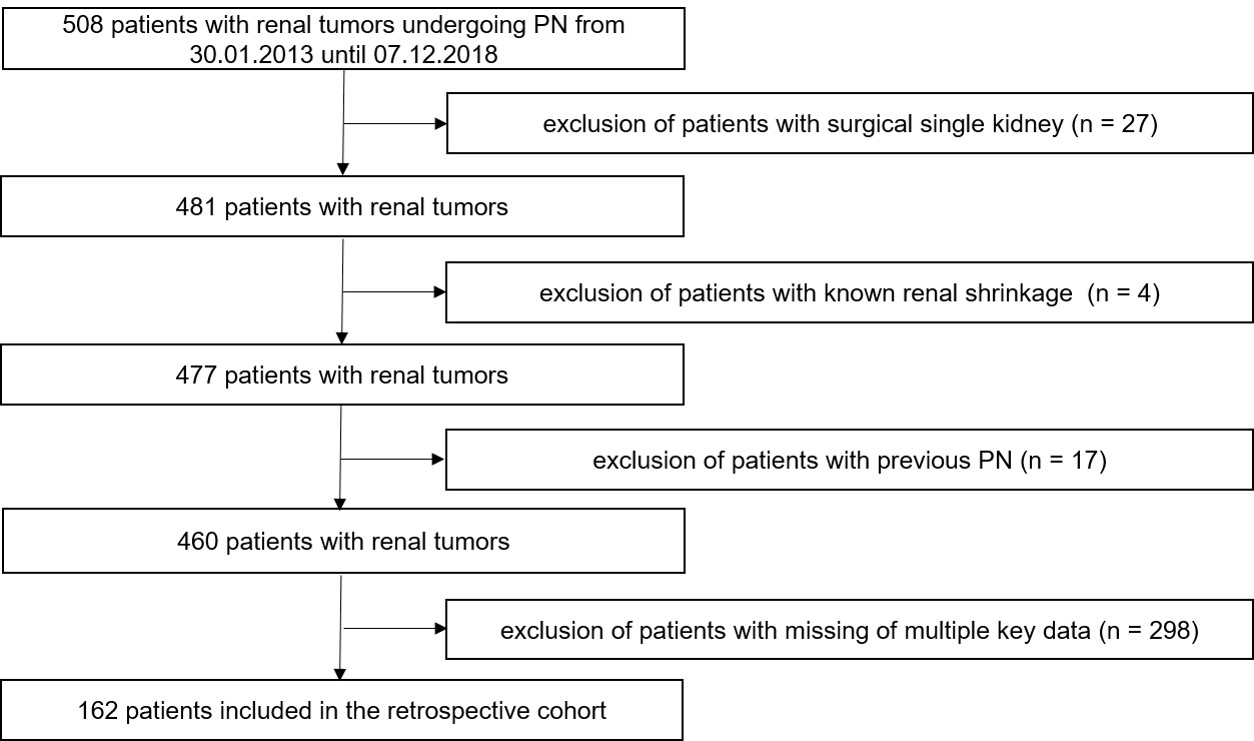

Supplement: Supplementary file 1 — Supplementary Fig. 1: Flow chart of exclusion criteria and cohort size of the retrospective cohort [file 423_2024_3513_MOESM1_ESM.jpg]

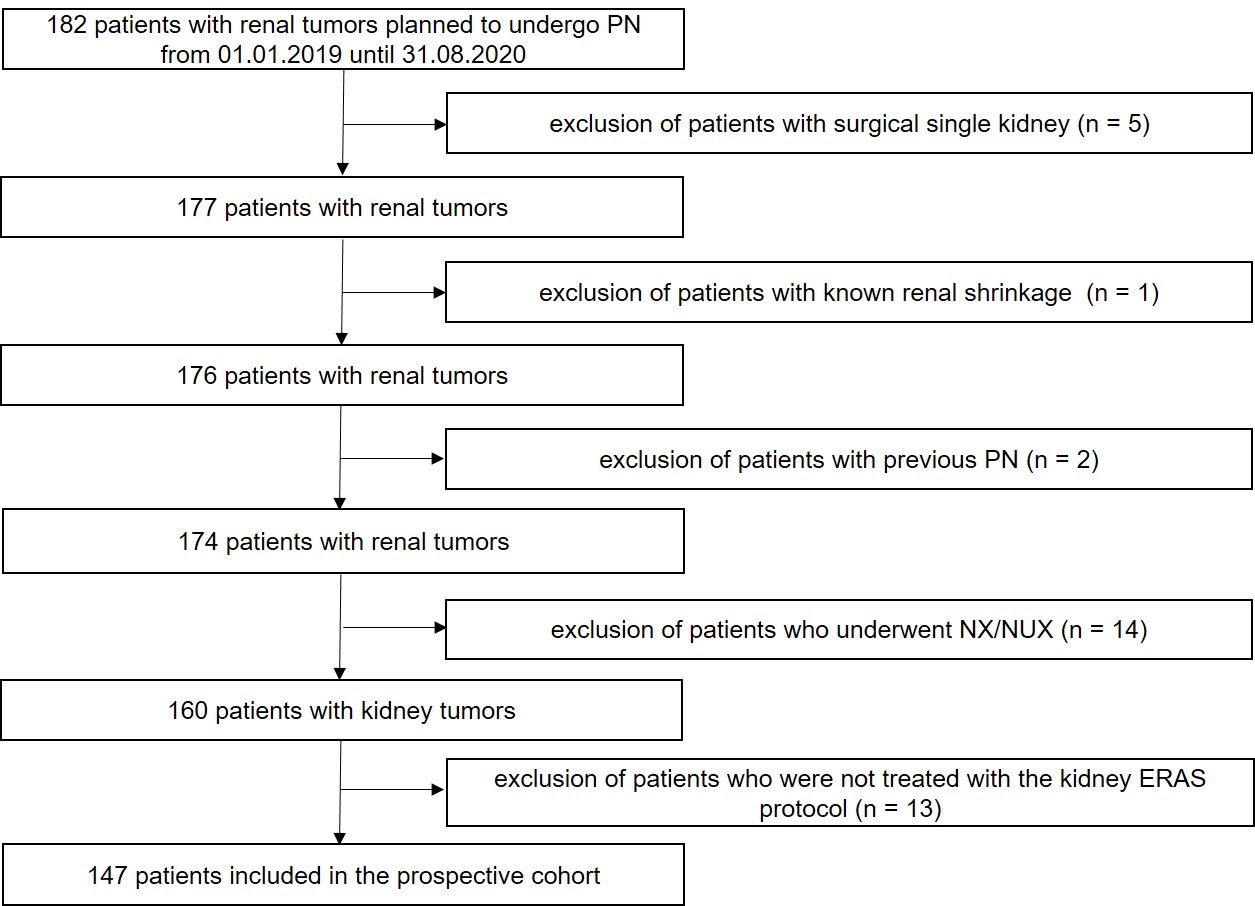

Supplement: Supplementary file 2 — Supplementary Fig. 2: Flow chart of exclusion criteria and cohort size of the prospective cohort [file 423_2024_3513_MOESM2_ESM.jpg]

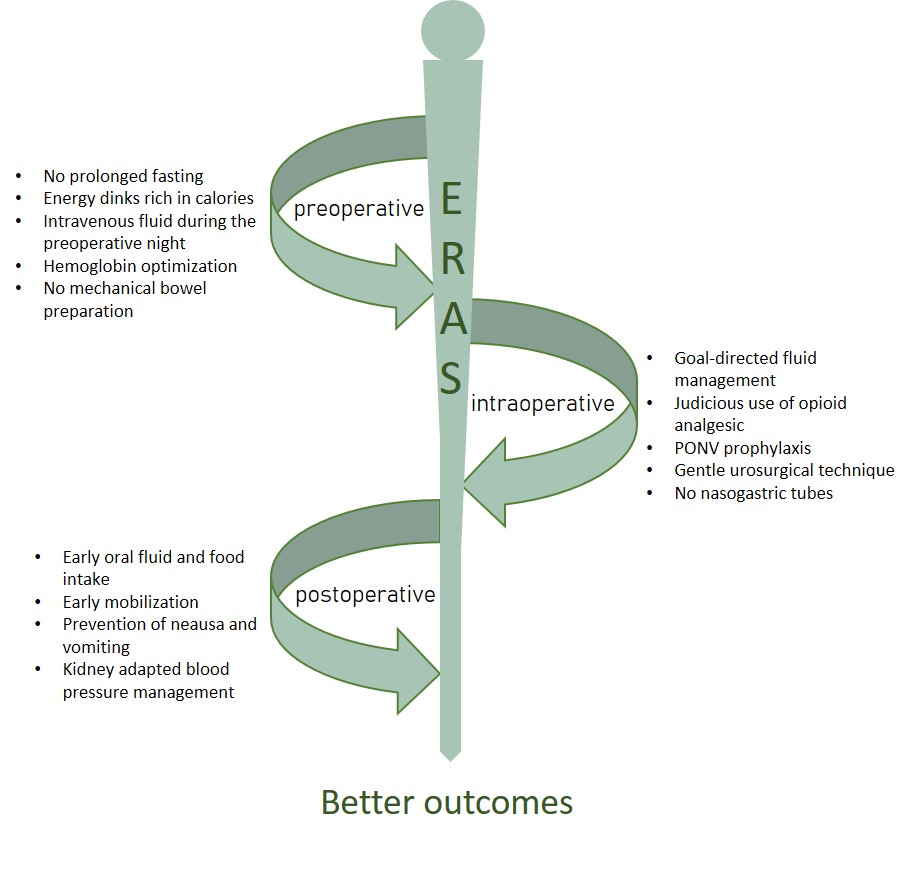

Supplement: Supplementary file 3 — Supplementary Fig. 3: Components of the kidney-adjusted ERAS protocol [file 423_2024_3513_MOESM3_ESM.jpg]
